# New insights into the distribution and spreading of the Asian walnut moth, *Garellamusculana* (Erschov, 1874) (Lepidoptera, Nolidae) in Europe with a focus on the Italian range

**DOI:** 10.3897/BDJ.11.e107609

**Published:** 2023-07-13

**Authors:** Penelope Zanolli, Davide Scaccini, Alberto Pozzebon

**Affiliations:** 1 Department of Agronomy, Food, Natural Resources, Animal and Environment, University of Padua, Legnaro, Italy Department of Agronomy, Food, Natural Resources, Animal and Environment, University of Padua Legnaro Italy

**Keywords:** alien species, pest, *Juglans*, early detection, *Erschoviella*, Italy

## Abstract

The Asian walnut moth, *Garellamusculana* (Erschov, 1874) (Lepidoptera, Nolidae) is an alien pest originating from Central Asia and is now spreading in Europe, attacking walnut trees. In this study, we updated the current distribution of *G.musculana*, focusing on the Italian range, where it was reported for the first time in 2021. Field surveys showed an extensive distribution of *G.musculana* in northern Italy, particularly in the Veneto Region. In this area, the Asian walnut moth developed on English and black walnut, attacking almost exclusively tree shoots. Based on current distribution data, further investigations are required in the nearby regions as well as in those that were less surveyed. Lastly, it is imperative to conduct more studies on insect biology and the impact on walnut production.

## Introduction

Alien arthropod species play a detrimental role in natural communities and ecosystems where they are introduced, as they can have high economic and environmental impact (e.g. Pyšek and Richardson 2010, Vaes-Petignat and Nentwig 2014, Early et al. 2016).

The genus *Juglans* is distributed in Asia, Europe and America and comprises approximately 22 species (Zhang et al. 2019). In Italy, the 4930 hectares of English walnut, *Juglansregia* L., 1753 (Fagales, Juglandaceae) produce about 15,490 tonnes of fruit (FAOSTAT 2021). Arthropod pests attack *J.regia* on both vegetative and reproductive plant parts and the main species belong to insects and mites, such as leaf gall mites, aphids, scales, flies, bark beetles and moths (Sharma et al. 2012). A novel insect species recently raised attention on the panorama of walnut moth pests, named the Asian walnut moth, *Garellamusculana* (Erschov, 1874) (Lepidoptera, Nolidae). *Garellamusculana* is an alien species that originated from Central Asia and is now spreading in Europe, developing on *Juglans* species like the English walnut, *J.regia* and the black walnut, *Juglansnigra* L., 1753 (Fagales, Juglandaceae) where larvae feed on shoots, buds and fruits (EPPO 2005, Bostancı et al. 2019, Bozkurt et al. 2020, Bostancı et al. 2021, EPPO 2021a). Previous records state *Prunusdulcis* (Miller) (Rosales, Rosaceae) and *Populus* spp. (Salicales, Salicaceae) as host plants for this pest (Esonbaev et al. 2020, Robinson et al. 2023), but these plant taxa still require confirmation, especially in new invasion areas.

*Garellamusculana* old records cover territories of Afghanistan, Kazakhstan, Kyrgyzstan, Tajikistan, Turkmenistan, Uzbekistan and the north of Iran, which likely represent the native area of the moth and it probably also occurs in China and Pakistan (Fibinger et al. 2009, EPPO 2021b, Lepiforum.org 2023, Fig. 1). Notably, records of *G.musculana* in the Oriental Region and Eastern Palaearctic require verification due to the presence of its eastern closely-related taxon, *Garellaruficirra* (Hampson, 1905) (Lepidoptera, Nolidae), with which it may be misidentified (Scaccini et al. 2023). Recent records of the Asian walnut moth identify this pest in the Kashmir Valley, India at least from 2011 (Khan et al. 2011), while for Eastern Europe and the Middle East in Crimea and United Kingdom since 2008 (Sviridov 2008, CABI 2022), in Turkey since 2015 (Yıldız et al. 2018), in Bulgaria since 2016 (Beaumont 2018), in Romania since 2018 (Bostancı et al. 2021) and in southern Russia and Italy mainland since 2021 (Fibinger et al. 2009, EPPO 2021b, Romanchuk and Kolesnikov 2021, Scaccini et al. 2023, Fig. 1). The report for the UK in 2008 (CABI 2022) needs to be checked further, since it was not reported in the other published material we checked.

Although for some countries, *G.musculana* findings were occasional, for others, the Asian walnut moth is more frequently detected (e.g. Yoğurtcu et al. 2018, Yoğurtçu and Kaçar 2018, Bostancı et al. 2019, Yıldız and Ayberk 2019, GBIF 2023) and, in some areas, it is a known pest that affects walnut production (Orozumbekov and Moore 2007, Sangov 2015, Yoğurtcu et al. 2018, Bragard et al. 2021, Yoğurcu and Kaçar 2022).

This study aimed to assess the current distribution of *G.musculana* in Italy after its discovery in 2021, mainly by ad hoc field surveys in the Italian territory, focusing on the area where the species was found for the first time. It is shown that, despite the recent first records, this species is more common and widely distributed in this country than previously expected, possibly representing a new threat to walnut cultivation.

## Material and Methods

### Data mining and field surveys

The current distribution of the Asian walnut moth at a global scale, with a focus on Europe and Italy was derived from accurate literature research coupled with field surveys in the Italian territory. The online research was performed searching for information on Asian walnut moth distribution in articles, books, reports, theses, dissertations, conference proceedings, unpublished reports, plant protection database (e.g. EPPO), web fora and in the material present in the reference lists of the previous documents. For this data mining, material in all the available languages was used and research keywords also included common names, previous scientific names and synonyms and erroneous terms that refer to *G.musculana* (see Fibinger et al. 2009, Scaccini et al. 2023).

Furthermore, to investigate the current distribution of *G.musculana* in Italy, investigations were conducted on main host plants during the growing season up to December, in both orchards and wild walnut plants found in hedgerows or private gardens. This monitoring activity was conducted in 2022 by searching for *G.musculana* symptoms, looking at both plant shoots and fruits and on trunk cracks, crevices and under loose bark searching for pupal cocoons (EPPO 2005, Yoğurtçu and Kaçar 2018, Bostancı et al. 2019, Bostancı et al. 2021). Field surveys were conducted in 151 geolocated points, most of them covering the Veneto Region (120 points) with particular reference to the lowlands and in the area close to the first findings in 2021 (Scaccini et al. 2023). Moreover, some additional points were monitored in Lombardy (9), Friuli-Venezia Giulia (2) and Emilia Romagna (20) Regions. In the Veneto Region, almond and poplar trees were also examined in two and three sites respectively where walnut was also present. Each surveyed point was georeferenced and the information was collected in a database. At each geolocated point, the elevation was also recorded. Further information on pest occurrence was collected directly from the farmers and technical advisers we contacted for field surveys. Living individuals and remains of *G.musculana* found in the field were taken to the laboratory to confirm the identification.

### Species identification

Insect identity was confirmed morphologically according to Fibinger et al. (2009) and Scaccini et al. (2023). Larvae were morphologically checked to the family level for their external characters following Martinat (1977). For larvae, species identification considered the observations previously reported on *G.musculana* and was confirmed through the DNA-barcoding cytochrome c oxidase I (COI) analysis as described in Scaccini et al. (2023).

### Distribution map

The Italian distribution map was built with QGIS (v. 3.4.2-Madeira) using a raster layer file of Italian regions retrieved from Geoportale Nazionale (2023), projected in WGS84. On the map, findings were reported with respect to *G.musculana* presence, with each geolocated point considered as one of the following categories: (i) absence of the Asian walnut moth (i.e. where symptoms or specimens were not found), (ii) occurrence of the pest by the presence of symptoms and (iii) presence confirmed, when symptoms were found together with specimens that were identified. While building the map, findings retrieved from the online research were included. Infested trees were classified according to the plant species, *J.regia*, *J.nigra* or other plants.

## Results

**Figure 1.**
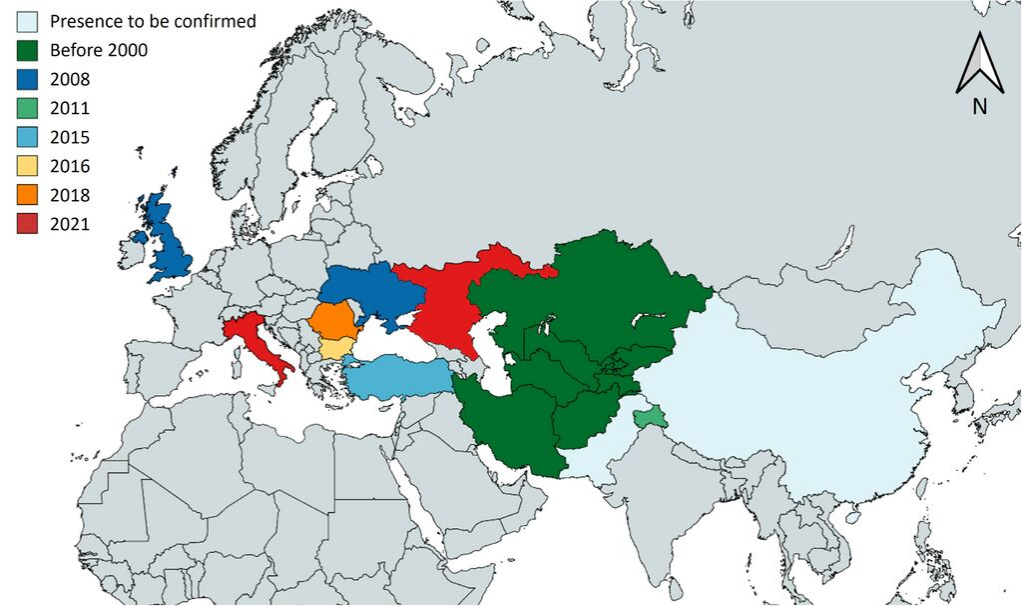
Global distribution of *Garellamusculana* in the native range (Central Asia, dark green) and in the invaded range retrieved from bibliographic data, by year of first detection. Base map from https://www.mapchart.net/italy.html.

**Figure 2.**
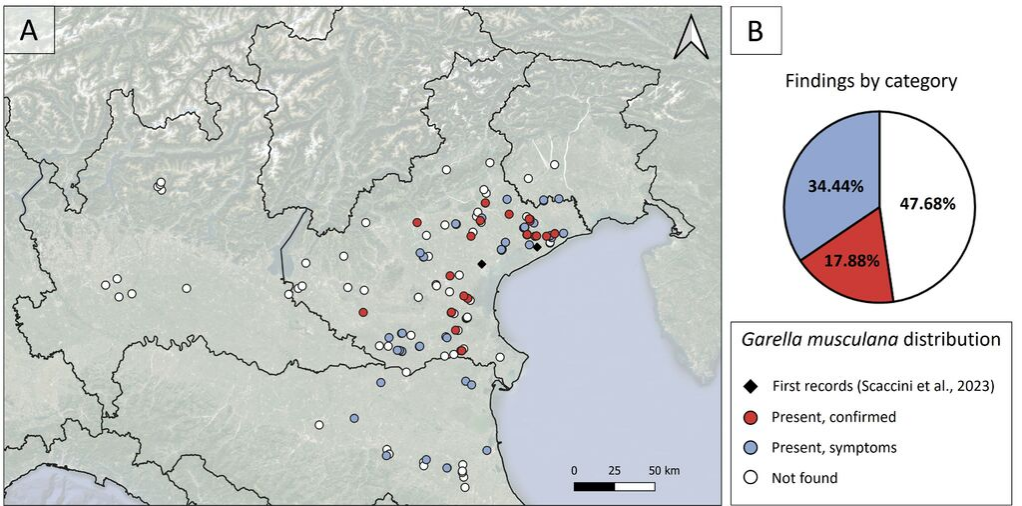
*Garellamusculana* in Italy. (A) Present distribution of *G.musculana* in Italy including the first two records for the country, also considering sampling points with no records. (B) Pie chart representing the amount of the three categories, based on original data appearing in the map.

Asian walnut moth distribution data, used to describe its global distribution, were included in the map in Fig. 1. In Italy, data mining ended in only one case of moth occurrence, an adult collected in a private garden in Vigonza, Padua Province, on 30 March 2022 (Forum Natura Mediterraneo 2022). *Garellamusculana* presence covered 79 points of the 151 surveyed, mostly in the Veneto Region (70, 58.33% of sampled points for this region), with some records in Emilia Romagna (9, 45.00% of regional points), where symptoms potentially ascribed to *G.musculana* were observed. This species was not recorded in Lombardy or Friuli-Venezia Giulia Regions. In total, 52.32% of the points resulted in infestation by the Asian walnut moth (Fig. 2). The altitudes at which *G.musculana* was recorded ranged from sea level up to 99 m a.s.l., with an average of 11.49 ± 2.26 m a.s.l. The dataset with the detailed distribution data is available in Suppl. material 1.

**Figure 3.**
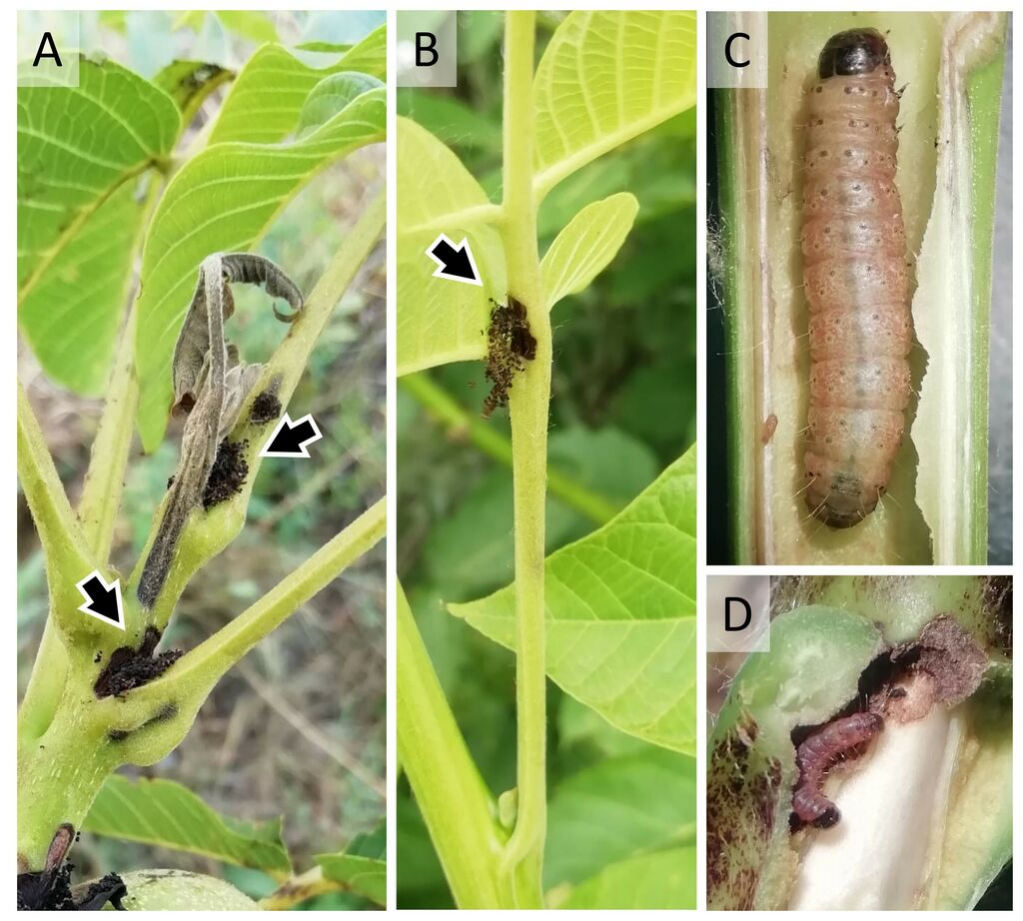
Damage by *Garellamusculana* of: (A) a 1-year-old *Juglansregia* shoot and (B) on a leaf. The arrows indicate the presence of larval frass and seta at the gallery entrance. (C) A mature larva in the gallery of a *J.regia* shoot and (D) a small larva in the entrance of its gallery in the leaf axils, opened to show the insect inside (Veneto Region, 2022). Photo credit: D. Scaccini.

Concerning host plants, the Asian walnut moth was collected from *J.regia* (54.23% of cases attributed to *J.regia*) and *J.nigra* (12.50% of *J.nigra*), while no records were shown for poplar or almond trees. Shoots of infested trees were yellowing, wilting, desiccating and excavated by the larva, with the presence of dark frass and seta at the entry hole, which is often on the leaf axil (Fig. 3A). Usually, a single larva inhabits the gallery (Fig. 3C, D). In some cases, similar symptoms were found even on the leaflet or leaf petioles, where only small larvae dig in plant tissues given their small size (Fig. 3B). Besides, in two cases, only *G.musculana* attack was reported on *J.regia* fruits.

*Garellamusculana* findings reported here refer to walnut orchards (87.34% of the finding points), isolated walnut trees and hedgerows (10.13%) and private gardens (2.53%). Despite in the aforementioned case of an adult found in a private garden, all the other observations refer to immature forms, with larvae found from the end of June to the first ten days of September. In some cases, pupae were also found on trunks in loose bark crevices.

## Discussion

Despite the first assessment by EPPO (2022) referring to the Asian walnut moth as transient for the Italian territory, this information should be updated while considering the results reported in this study. The current distribution of the Asian walnut moth suggests tracing back its establishment in the Italian territory as early as a few years before the first findings in 2021. Further investigations are, however, required.

In this study, points on a map where *G.musculana* presence was confirmed are separated from those where only symptoms were observed. Indeed, sometimes *G.musculana* symptoms may be misidentified with those of *Zeuzerapyrina* (Linnaeus, 1761) (Lepidoptera, Cossidae), an indigenous carpenter moth that develops on twigs and small branches of many host plants (Yakovlev 2012). This and other carpenter moth species are, in fact, common, serious pests of agriculture and forestry that affect both native and introduced plant species worldwide (e.g. Yakovlev 2012, Tavares et al. 2020, Yadav et al. 2020, Scaccini et al. 2021). However, the hypothesis of moth misidentification is unlikely since shoots damaged by the larvae of *Z.pyrina* differ from those hosting *G.musculana* by the presence of sap emission for *Z.pyrina* and not for *G.musculana* and more seta and frass/faecal pellets on the entrance of galleries in the case of the latter. In the surveyed sites, plant infestation by the Asian walnut pest was more relevant than those observed for carpenter moths, which, in turn, concerned only a few sites. Furthermore, in the case of moth larvae findings in walnut shoots, *Z.pyrina* larvae cannot be confused with those of *G.musculana* since they are yellow with dark spots, differing from those of the alien pest.

The Asian walnut moth damage on plants was consistent with those reported by previous studies, with typical damage on shoots (e.g. EPPO 2005, Bostancı et al. 2019, Bostancı et al. 2021, Scaccini et al. 2023) and attacks on fruit that, however, were only sporadic in the surveyed territory. Damage was observed on *J.regia* and *J.nigra* only, but it should be considered that more time has been devoted to these species in the sampling effort. In the case of pupae, they were detected under loose bark and in other refuges on the trunk, especially for old walnut trees that have more of these refuges. The first adult of the season was found in late March, confirming what was previously reported, with first adult appearance known from the beginning of April (EPPO 2005, GBIF 2023). However, *G.musculana* biological features and phenology in Italy are not yet well understood.

## Conclusion

Field surveys showed that *G.musculana* is widely distributed in north-eastern Italy and particularly in Veneto Region. Future studies should be dedicated to the investigation of biological features of this pest and to better understand its distribution. Finally, the impact on cultivated plants has to be researched in Italian walnut orchards in order to plan suitable management tactics against this alien pest.

## Acknowledgements

We are grateful to people who helped us during field surveys and collected more data on *G.musculana* distribution in Italy, especially Cesare Bendandi, Eugenio Cozzolino, Luca Fagioli, Antonio Fiorin and Alessio Signori, the farmers and other technicians who hosted our surveys in the orchards. We thank Francesco Sanna (TESAF – University of Padua) and Filippo Simoni (DAFNAE – University of Padua) for helping during walnut inspections in the field. We would also like to thank Matteo Marchioro, Alberto Mele and Enrico Mirandola (DAFNAE – University of Padua) for their help in building the distribution maps. The present work was supported by Regione Veneto U.O. Fitosanitario.

## Supplementary Material

BCC30E5C-ED95-53CA-A0F2-AE075F6A0C91 10.3897/BDJ.11.e107609.suppl1 Supplementary material 1 Occurrence of *Garellamusculana* in Italy Data type Occurrences Brief description Occurrence of *Garellamusculana* in Italy, comprising of surveyed sites and those from data mining and previous published material. For "Instar" column: ad. = adult/s, la. = larva/e, pu. = pupa/e. File: oo_860354.xlsx https://binary.pensoft.net/file/860354 Scaccini D., Zanolli P., Pozzebon A.
